# Miscellany

**Published:** 1893-03

**Authors:** 


### Miscellany.

### WORLD'S COLUMBIAN EXPOSITION.

*The World's Congress Auxiliary.—Department of Moral and Social Reform Congresses.—The International Congress of Charities, Correction, and Philanthropy.*

The Congress will meet in the City of Chicago on Monday morning, June 12, 1893, at ten o'clock, in a hall to be hereafter announced.

#### SECTIONS OF THE CONGRESS.

(1) The Public Treatment of Pauperism; (2) The Care of Neglected, Abandoned, and Dependent Children; (3) The Hospital Care of the Sick, the Training of Nurses, Dispensary Work, and First Aid to the Injured; (4) The Commitment, Detention, Care, and Treatment of the Insane; (5) The Prevention and Repression of Crime, and the Punishment and Reformation of Criminals; (6) The Organization and Affiliation of Charities in Countries, States, Cities, Towns, and Villages, and Preventive Work Among the Poor; (7) The Introduction of Sociology as a Special Topic of Investigation and Instruction in Institutions of Learning.

The Committee of Organization will add sections covering other fields of philanthropic work if there should seem to be a sufficiently general demand for them.

FREDERICK H. WINES,
JOHN G. SHORTALL,
MRS. J. M. FLOWER,
*Committee of Organization.*
NATHANIEL S. ROSENAU,
*Secretary to the Committee.*

OFFICERS OF SECTIONS.

SECTION 1.—The Public Treatment of Pauperism; Chairman, Ansley Wilcox, of New York, Chairman Executive Committee, Charity Organization Society of Buffalo; Secretary, John H. Finley, of Illinois, President of Knox College, Galesburg.

SECTION 2.—The Care of Neglected, Abandoned, and Dependent Children; Chairman, Mrs. Anna Garlin Spencer, of Rhode Island, Providence; Secretary, Charles L. Birtwell, of Massachusetts, Secretary Children's Aid Society, Boston.

SECTION 3.—The Hospital Care of the Sick, the Training of Nurses, Dispensary Work, and First Aid to the Injured; Chairman, Dr. John S. Billings, Surgeon U. S. A., Washington, D. C.; Secretary, Dr. Henry M. Hurd, of Maryland, Superintendent Johns Hopkins Hospital, Baltimore. Sub-section on the Training of Nurses; Chairman, Miss Isabel A. Hampton, Superintendent Training School for Nurses, Johns Hopkins Hospital, Baltimore.

SECTION 4.—The Commitment, Detention, Care, and Treatment of the Insane; Chairman, Dr. G. Alder Blumer, of New York, Superintendent State Hospital for the Insane, Utica; Secretary, Dr. A. B. Richardson, of Ohio, Superintendent Asylum for the Insane, Columbus.

SECTION 5.—The Prevention and Repression of Crime, and the Punishment and Reformation of Criminals; Chairman, Charlton T. Lewis, of New York, President State Prison Association, New York City; Secretary, John L. Milligan, of Pennsylvania, Secretary National Prison Association, Allegheny.

SECTION 6.—The Organization and Affiliation of Charities in Countries, States, Cities, Towns and Villages, and Preventive Work Among the Poor; Chairman, Daniel C. Gilman, of Maryland, President Johns Hopkins University, Baltimore; Secretary, ———

SECTION 7.—The Introduction of Sociology as a Special Topic of Investigation and Instruction in Institutions of Learning; Chairman, E. Benjamin Andrews, of Rhode Island, President Brown

University, Providence; Secretary, Amos G. Warner, of California, Professor of Economics, Leland Stanford, Jr., University; present address, Washington, D. C.

PROGRAMME OF SESSIONS AND SUBJECTS.

1. The National Conference of Charities and Correction will hold its twentieth annual meeting from June 8th to 11th inclusive.

2. The National Prison Association will also hold its annual meeting during the week preceding the International Congress.

3. The International Congress of Charities, Correction and Philanthropy will hold its sessions from June 12th to 18th inclusive.

FIRST DAY, Monday, June 12, 1893.—10.00 to 11.15 A. M., Opening Exercises; 11.15 A. M. to 12.00 M., Oration (subject to be announced); 12.00 M. to 2.00 P. M., Luncheon and Conversazione; 2.00 to 4.00 P. M., Opening Meetings of the Sections; 8.00 P. M., General Session, Subject, The Public Treatment of Pauperism.

SECOND DAY, Tuesday, June 13, 1893.—10.00 to 10.30 A. M., Business Session; 10.30 A. M. to 12.30 P. M., Meetings of the Sections; 12.30 P. M., Visit to County and Presbyterian Hospitals; 8.00 P. M., General Session, Subject, The Organization and Affiliation of Charities in Countries, States, Cities, Towns, and Villages, and Preventive Work Among the Poor.

THIRD DAY, Wednesday, June 14, 1893.—10.00 A. M. to 12.00 M., General Session, Subject, The Hospital Care of the Sick, the Training of Nurses, Dispensary Work, and First Aid to the Injured; 2.00 to 4.00 P. M., Meetings of the Sections; 8.00 P. M., General Session, Subject, The Care of Neglected, Abandoned, and Dependent Children.

FOURTH DAY, Thursday, June 15, 1893.—10.00 to 10.30 A. M., Business Session; 10.30 A. M. to 12.30 P. M., Meetings of the Sections; 1.00 P. M., Visits to Charitable and Correctional Institutions of Chicago and Vicinity; 8.00 P. M., General Session.

FIFTH DAY, Friday, June 16, 1893.—10.00 A. M. to 12.00 M., General Session, Subject, The Introduction of Sociology as a Special Topic of Investigation and Instruction in Institutions of Learning; 12.00 M. to 12.30 P. M., Descriptive Paper on the Bureau of Charities and Correction of the World's Columbian Exposition; 8.00 P. M., General Session, Subject, The Prevention and Repression of Crime and the Punishment and Reformation of Criminals.

SIXTH DAY, Saturday, June 17, 1893.—10.00 to 10.30 A. M.,

Business Session; 10.30 A. M. to 1.30 P. M., General Session, Subject, the Commitment, Detention, Care, and Treatment of the Insane; 3.00 to 4.00 P. M., Closing Meetings of the Sections; 8.00 P. M., General Session, Subject, The Apprehension and Detention of Prisoners, and the Bertillon System of Registering and Identifying Criminals.

SEVENTH DAY, Sunday, June 18, 1893.—Morning, Religious Exercises with Sermon Germane to the Work of the Congress; Evening at 8.00 P. M., General Session, Essay, The Relation of the Kindergarten to Pauperism and Crime; Address, Manual Training in its Effects on Character and the Reduction of Pauperism; 9.10 P. M., Closing Exercises.

Address communications to Nathaniel S. Rosenau, Secretary, World's Congress Headquarters, Chicago, Ill., U. S. A.

### SECTION THREE ON THE HOSPITAL CARE OF THE SICK, THE TRAINING OF NURSES, DISPENSARY WORK, AND FIRST AID TO THE INJURED.

One of the series of International Congresses to be held in Chicago in 1893 is to be devoted to the subjects of Charities, Correction and Philanthropy, and the third section of this is to consider all matters relating to the Hospital Care of the Sick, the Training of Nurses, Dispensary Work, and First Aid to the Injured. The Committee of Organization of the Congress has appointed Dr. John S. Billings, Surgeon U. S. Army, as Chairman of this Section, and Dr. Henry M. Hurd, Superintendent of the Johns Hopkins Hospital, in Baltimore, as its Secretary, and has authorized and requested them to complete its organization, to extend invitations, and to prepare a programme for its work. Miss Isabel Hampton, Superintendent of the Training School for Nurses of the Johns Hopkins Hospital, has been appointed Chairman of that part of the work of the section which relates to the training of nurses.

This section will hold five sectional meetings of about two hours each, commencing June 12, 1893, and will also have charge of one of the general sessions of the Congress, viz., that held on the morning of June 14th.

It is desired that this shall be a truly international gathering for conference on the subjects allotted to this section, and all who are interested in hospitals, in training of nurses, in dispensaries, or in first aid to the injured, are cordially invited to be present, to contribute papers, and to take part in the discussions.

The papers and proceedings will probably be printed as a separate volume, and it is hoped that this will represent the best methods and the best work in each of these departments in all parts of the world.

The following are suggested as subjects for special consideration in papers to be prepared: (1.) Hospital Organization—Governing Bodies—Relations of the Medical Staff and of Nurses' Training Schools. (2.) Hospital Finances—Means of Support—Mode of Keeping Accounts—Cost. (3.) Plan and Construction of recently built General Hospitals, embodying the latest improvements. (4.) Relations of Hospitals to Increase of Knowledge, to Medical Education, and to the Medical Profession. Hospital Records, Statistics, and Reports. (5.) Pay Patients in Hospitals. (6.) Isolating Wards and Hospitals for Contagious Diseases. (7.) Hospital Diets, Dietaries, Kitchens, etc. (8.) Hospital Amphitheatres and Operating Rooms. (9.) Hospital Laundries and Disinfecting Establishments. (10.) Army and Navy Hospitals—Emergency Hospitals in Time of Epidemics—Temporary and Movable Hospitals. (11.) Small and Special Hospitals, Cottage Hospitals, School Hospitals, Private Hospitals, Sanatoriums, etc. Convalescent Hospitals, and what to do with Incurables. (12.) History and Present Condition of Hospitals in the Large Cities. (13.) Training Schools for Nurses—see special circular. (14.) Dispensaries—Relations to the Public and to the Medical Profession. Dispensary Records. (15.) First Aid to the Injured. Associations for best means of popular instruction in, and its place in, General Education.

Persons desiring to present papers, or to share in the discussions of this section, are requested to communicate with the Secretary at once. The period of time allotted for the preparation of the programme is necessarily brief, and it is essential that all who are willing to assist in this work should act promptly.

JOHN S. BILLINGS, M. D., *Chairman.*
HENRY M. HURD, M. D., *Secretary.*

Address all communications to Dr. Henry M. Hurd, the Johns Hopkins Hospital, Baltimore, Md.

